# Living Well with Dementia: Feeling Empowered through Interaction with Their Social Environment

**DOI:** 10.3390/ijerph20126080

**Published:** 2023-06-08

**Authors:** Berit Ziebuhr, Michele Zanasi, Yolanda Bueno Aguado, Raquel Losada Durán, Tom Dening, Isabelle Tournier, Kristina Niedderer, Ana Diaz, Diana Druschke, Rosa Almeida, Vjera Holthoff-Detto

**Affiliations:** 1 St. Hedwig Kliniken Berlin, Alexianer Krankenhaus Hedwigshoehe, Berlin 12526, Germany; 2 Fundación INTRAS, INTRAS Neuropsychology Clinic, 47016 Valladolid, Spain; 3 Fundación INTRAS, RDi Projects Department, 47016 Valladolid, Spain; 4 Mental Health & Clinical Neurosciences, School of Medicine, University of Nottingham, Nottingham NG7 2TU, UK; 5 Department of Design, Manchester School of Art, Manchester Metropolitan University, Manchester M15 6BR, UK; 6 Department of Psychology, Laboratoire Cliniques Pathologique et Interculturelle, Université Toulouse 2 Jean Jaurès, 31058 Toulouse, France; 7 Alzheimer Europe, 1417 Luxembourg, Luxembourg; 8 Center for Evidence-Based Healthcare, University Hospital Carl Gustav Carus, Carl Gustav Carus Faculty of Medicine, University of Technology Dresden, 01307 Dresden, Germany; 9 Department of Psychiatry and Psychotherapy, Carl Gustav Carus Faculty of Medicine, University of Technology Dresden, Alexianer Krankenhaus Hedwigshoehe, 12526 Berlin, Germany

**Keywords:** dementia, usefulness, empowerment, self-determination, shared decision-making

## Abstract

This study was designed to advance our understanding of how feelings of empowerment in people living with dementia still residing at home can be promoted. We conducted qualitative interviews with 12 participants with mild-to-moderate stages of dementia in Germany and Spain as part of a European study on mindful design for dementia. A qualitative thematic content analysis was performed to elicit the key features of the experience reported by the interviewees. Three overarching categories were identified: the first category ‘experiencing changes in personal life and coping with changes in life’ covered losses and coping strategies; the second category ‘retaining a sense of usefulness‘ included social participation and the need for activities with others; the third category ‘feeling empowered’ covered reflections on lifetime achievements, accomplishments in the present life, being in control and self-worth. Participants placed a strong emphasis on continuity and on the importance of making active decisions and meaningful social contributions. Empowerment within the person living with dementia was achieved through their interactions with their social environment, including the significance of communication about their needs and wishes and enabling shared decision-making and interactions with others in reciprocity.

## 1. Introduction

Several factors are consistently associated with psychological and physical health into advanced age. Staying healthy and maintaining independence, retaining control over one’s life, having close relationships, having insightful experiences and engaging in purposeful activities with others, and feeling useful all contribute to one’s well-being and sense of personal cohesion in advanced age [1,2,3]. These factors are also highly relevant for people living with dementia (PwD). Dementia can affect many aspects of a person’s life, such as their feelings about themselves, their relationships with very close people and their social interactions, and how they wish to continue their lives with health deficits and which support they wish to rely on. Two-thirds of people diagnosed with dementia desire to live in their own homes and want to continue to live as independently as possible [4].

The number of PwD in European countries is estimated to be almost 10 million [5]. A recently conducted survey in Germany revealed that 18% of the population older than 80 years is affected by dementia and 70% still live at home [6]. In 2050, dementia will affect 3.4% of the population in Germany and 3.9% in Spain [7]. However, the complex and chronic progressive nature of dementia leads to changes in cognitive abilities, autonomy, role function and personal worries about the future. Dementia is portrayed and very often associated with an immediate inability to communicate, to understand and to make one’s own choices regardless of the clinical stage. The clinical course of dementia includes differing stages, starting with a period of early symptoms, then moderate symptoms and, in the last years of life, severe dementia. PwD have a strong wish in the early and moderate stages of the disease and, more tentatively, in the later stages to be heard, to make their own decisions, to actively participate in social life as equal citizens and they do not want to rely on carers to speak on their behalf [8,9]. Programs were shown to support PwD in their self-determination and decision-making by increasing the awareness of their wishes, thereby also reducing the perceived extent of social isolation [10] and stigma [11,12]. Many of these programmes are based on a model developed by Kitwood, in which the personhood of PwD is central and behaviour is a reflection of their personality and coping strategies are a result of their life history, social and physical environment, and functioning of their brain affected by dementia [13]. In a programme developed by the WHO for carers (iSupport), these elements are integrated into exercises and are based on therapeutic approaches that showed some beneficial impact, including elements of cognitive behavioural therapy, such as psychoeducation, behavioural activation, cognitive reframing and some problem-solving elements [14,15]. However, the implementation of these programmes and subsequent research is insufficient, and investigations revealed that PwD still do not feel like active participants relative to their situation in their daily living [9]. Empowerment in PwD is a term associated with informational, educational and psychosocial support necessary to promote a sense of identity, usefulness, control and self-worth [16]. A group involving researchers and experts by experience worked on a definition of the term ‘empowerment in PwD’ and agreed on core components of empowerment relevant to PwD [9]. Empowerment is described as a dynamic confidence-building process between PwD and their social environment whereby PwD are respected, have a voice, are heard and are involved in making decisions about their lives. Furthermore, they have the opportunity to create change through access to appropriate resources [9].

First-person accounts revealed how well PwD are able to explain their conditions, wishes and needs [17], and we chose to conduct exploratory qualitative interviews for the study presented here. We were especially interested in learning more about situations in participants’ daily lives and social engagement that had the potential for the interviewees to retain a sense of usefulness and empowerment and to specify a more nuanced understanding of what they felt could support their feeling of self-worth. Therefore, we chose PwD still living at home in Germany and Spain and conducted in-depth qualitative interviews with participants with mild-to-moderate stages of dementia as part of the European MinD project. Participating interviewees with dementia were encouraged to describe their role and feelings in interactions with others, to report how specific situations made them feel, what was important to them, and what was or could be supportive. The MinD project also made it possible to compare the results of in-depth individual interviews obtained in two different European countries, permitting a comparison of the narratives given by PwD living in Germany and Spain as part of the analyses.

The Mind project is a European project on mindful design in dementia (‘MinD—Designing for People with Dementia’) [18]. The MinD project has published guidelines for the use of design to support the psychosocial well-being of PwD [19]. The project included the investigation of the challenges and opportunities of increasing the subjective well-being and social engagement for PwD through design from a mindfulness perspective and to use design and mindfulness to help people with early-to-mid-stage dementia to engage in social contexts and meaningful activity to improve their psychosocial well-being [19].

## 2. Methods

### 2.1. Participants

We undertook exploratory qualitative interviews with people still living in their homes with mild-to-moderate dementia in Germany and Spain using a semi-structured interview guide. Researchers in Berlin, Dresden and Valladolid conducted the interviews. We chose a cross-national analysis of qualitative patient insights in these two countries so that the findings might be more transferable, and both samples were convenience samples from dementia facilities in contact with the participating research groups. We approached participants by visiting local Alzheimer Association groups (Germany) and memory disorder clinics (Germany, Spain). Inclusion criteria were the onset of the disease later than age 65, having been diagnosed with mild-to-moderate dementia; being free of other psychiatric comorbidities, being a native speaker of German or Spanish, and living at home. In both countries, an experienced old-age psychiatrist established the full capacity to consent in a clinical evaluation. The Medical Ethics Review Committee at the Aerztekammer Berlin approved the study in July 2016 (number 30/16). The Castilla y León Regional Public Service for elderly care and the recruitment entity INTRAS Ethical Board approved the present study in September 2016.

### 2.2. Procedure

Three interviewers experienced in working with older people and specifically people with dementia and who were trained for interviewing and analyzing qualitative interview data (a native German speaker (B.Z.), a native Spanish speaker (R.L.) and a bilingual speaker (M.Z.)) conducted the semi-structured interviews. Interviewers in both countries met with the individual interviewees in a calm room at the memory clinic or in their own homes. Carers were not in the room while the interviews were conducted. All three researchers received expert supervision from an experienced qualitative researcher (D.D.). Information sheets outlined the study for the participants. Verbal and written informed consent was obtained from all participants.

### 2.3. Data Acquisition

Data collected included demographic characteristics and a semi-structured interview. The participant characteristics included age, gender, education, living situation and type of dementia. Educational level was assessed in three categories: university level (high), >10 years (medium) and <10 years (low).

The semi-structured interview guide was developed in a collaboration between the study team and an interdisciplinary team including clinical experts, as well as experts in design and experts by experience (European Working Group of People with Dementia and local groups in Germany and the UK) in an English version, which was then translated by native speakers to provide German and Spanish versions. Experts by experience commented on the German and English versions of the schedule to ensure that questions were worded in accessible language. Questions sought to elicit what people with dementia experience as changes in their personal lives and what made them feel useful, empowered and socially active. The interview addressed and discussed managing, coping and decision-making (a) in daily life (activities of daily living), (b) during social activities, and (c) in what situations the interviewees experienced as empowering and supportive for their well-being and feeling of self-worth. The researchers asked the interviewees how these situations made them feel and what would have been helpful for them at that moment. The questions were chosen to provide insight into people’s perspectives of their current situation, their feelings associated with aspects of their lives and their wishes for support.

The interview schedule provided orientation for the interviewer and still allowed for thematic digressions. Questions were open-ended to promote an exchange of information and opinions that was as unaffected as possible. The schedule provided additional minor questions and these were used if participants only addressed parts of the corresponding subject area. Interviewers started by asking the participants with dementia to imagine and describe a typical day in their lives. From there, the interviewer followed the interview guideline, which, e.g., specifically asked the interviewees about their feelings, as well as what made them experience a sense of usefulness and empowerment or what would have supported those feelings in the situations they recalled during the interviews.

Interviews lasted between 60 and 90 min, were audio-recorded and transcribed verbatim, and were anonymized following protocols for secure data storage. Data were analyzed using unique and anonymous codes. The interviewing researchers and a supervising researcher (D.D.) met on a regular basis and data collection in Germany and Spain continued until no new subcategories and subcategory contents were being generated and data saturation had probably been achieved.

### 2.4. Data Analysis

We undertook thematic content analysis to elicit the key features of the experience reported by the interviewees using the qualitative content analysis of Mayring [20], which allows for both deductive and inductive creation of categories [21]. The interviewers of the German and Spanish research groups met personally on a regular basis to discuss the content analysis and the emerging categories, subcategories and subcategory contents. To achieve an understanding of the data, they developed a coding system for categorizing the participants’ answers, which allowed for the reduction of statements and the detection of groups of answers.

We used a mixed inductive–deductive approach, meaning that, on the one hand, already existing scientific knowledge was integrated into the structure of the interview guideline; on the other hand, new categories were formed based on both the German and Spanish interview data. Based on the content, all relevant text passages were extracted and put into their specific categories [21]. Missing but relevant categories were added inductively based on the transcribed material up to the point at which the system became saturated (e.g., no new categories and subcategories emerged) [21]. This method complies with the principle of openness within qualitative research. The transcripts and coding framework were entered into MAXQDA (2018) (VERBI Software GmbH, Berlin, Germany) to assist with the management of further qualitative data analysis. The three coders systematically applied the finalized coding tree to all transcripts. The citations were translated from German and Spanish into the English language by a native speaker for the purpose of publication.

## 3. Results

### 3.1. Demographic Data

Twelve people living with dementia participated in the qualitative interviews (Table 1). All participants had received an ICD-10 (International Classification of Diseases, Version 10) diagnosis of dementia in a mild-to-moderate stage in a specific diagnostic setting, such as a memory clinic or from a specialized general practitioner. The number of female participants, the level of education and the living situation were comparable between both countries. The mean age of the German interviewees was almost 8 years more than the Spanish group.

### 3.2. Interview Categories

The 12 interviews allowed for comprehensive insights into the world of the participants affected by dementia. From their statements, three main categories could be identified: The first category ‘experiencing changes in personal life and coping with changes in life’ covered changes in cognition and emotions, losses of familiar roles, autonomy, self-determination and social contacts, and adaptive and non-adaptive coping strategies. The second category ‘retaining a sense of usefulness‘ included subcategories such as social participation and the need for activities with others. The third category ‘feeling empowered’ covered reflections on lifetime achievements, accomplishments in the present life and being in control (Table 2). The contents supporting the categories and subcategories are outlined in Table 2. Selected quotes are used in the following text sections to illustrate the subcategory contents.

### 3.3. Experiencing Changes in Their Personal Life and Coping with Changes in Life

The changes perceived included things such as changes in memory and orientation or feeling more lonely and insecure in familiar situations.

*I feel so terribly lonely and that makes me suffer. I have never cried much in my life, but now I am so very close to tears.*

(DE-II-18)

*When I wake up in the morning I feel anxious when thinking of what I need to do even if it is nothing special, like getting myself prepared for the day.*

(DE-II-17)

Participants described a range of effects on their current daily lives, including a loss of autonomy and changes in the way that decisions are taken. They appreciated it when their caregiver supported them to retain independence and the feeling of being in control.

*Somehow, everything is too fast…it becomes difficult, because you simply cannot contribute to the conversation anymore…and I am glad to return home, where everything happens at my normal pace.*

(DE-II-07)

*My daughter helps me with the financial management but she does not ask me on what I spend my money.*

(ES-II-04)

They were aware of their tendency to withdraw from familiar situations, such as family reunions, and realized that external support for coping with increasing demands in their lives was necessary. However, they expressed their wish to cope with their lives in order to stay in control and maintain normal daily life situations.

*I put a lot of time trying to be as autonomous as possible, because I do not want to be a burden to others, although I know that the older I get the more dependent I will be.*

(ES-II-07)

Most participants reported a general lack of opportunities to be active in their familiar roles. They described that they missed activities within their families, e.g., cooking for them, preparing themselves (e.g., wearing something special) for others, or being called for support and assistance or asked for advice.

*We do not see each other much anymore. That used to be, when we were still able to assist our children in their garden and in the house.*

(DE-II-07)

*My children used to ask me for advice and for my help, but they do not call on me anymore.*

(DE-II-04)

Spanish participants attributed specific importance to their social contacts while attending church services and saw them as an important means of maintaining social interactions with friends. The majority of participants in both countries also described that they saw much less of family, friends or colleagues and that they stopped being invited. They attributed that fact to being less useful to others.

*I was the one to prepare the meal for the whole family, but now I am unable to do so. They do not come to visit anymore.*

(DE-II-03)

*We used to go on bicycle tours in a group of friends and I was the one in charge of deciding on the tour. Since I stopped planning the tour, they do not ask me to come along anymore.*

(DE-II-02)

Participants also expressed concerns for the future. Most reported that external support gave them a feeling of safety.

*If I have somebody nearby, so that I can call for help. Then I enjoy taking a shower.*

(DE-II-04)

However, they also described situations that made them feel dependent and that they were losing control.

*Holding her hand does not bother me, but it makes me feel so dependent. I do not appreciate that.*

(DE-II-07)

*She wants to take care of me too much and let me ask you: Do you picture me that dependent on others?*

(ES-II-04)

Although several participants described a feeling of uselessness, others showed considerable understanding and empathy for caregivers. Participants realized that caregivers were concerned about their safety and well-being and how much responsibility caregivers had to take on their behalf.

*We take each other’s hand so nothing can happen … and my wife has a feeling of safety.*

(*PwD referring to his caregiver*, (DE-II-07))

All participants reflected upon future situations and were concerned about the prospect of becoming an increasing burden to the caregivers.

*And it has to be worse for her, much worse than for me, I think, because I forget most of the things I am expected to do.*

(DE-II-08)

### 3.4. Retaining a Sense of Usefulness

When asked to describe what was important to their daily lives, the participants expressed the desire to put effort into maintaining usefulness and social contribution by being more active together with family members and friends and meeting with others on equal terms. However, most participants had strong experiences of being less indispensable than previously.

*I used to trim trees and now they let me do the weeding.*

(DE-II-07)

*I love to knit socks, but there is no one I could give them to.*

(DE-II-04)

*I used to cook for the family and now nobody comes to eat my meals.*

(ES-II-15)

When asked what contributed to feeling useful, all interviewees were able to give examples of how important the feelings of being needed, close to others and being a part of something were. Participants expressed the desire to maintain a social life with family, friends and old workmates and how that strengthened their feeling of inclusion and self-worth.

*I feel well, I cope with life, I feel good with myself, I go out with friends and can go to places I like.*

(ES-II-02)

*The feeling of being in the middle of a group of affectionate people who also like me is what I need.*

(DE-II-18)

*Well, we attend church where people meet and this year we have this celebration in Valladolid and there we will meet with many couples.*

(ES-II-06)

*Being present for another person makes me feel satisfied and needed. It makes me happy when people smile at me and ask for my support again.*

(ES-II-01)

*I like to invite my grandson with his girlfriend for lunch. They like to eat my meals and they also support me. They are very considerate grandchildren.*

(DE-II-15)

### 3.5. Feeling Empowered

A feeling of empowerment was described when participants reminded themselves of lifetime achievements (e.g., bringing up children and performing in a job), which preserved their identity and self-cohesion and strengthened their feelings of self-worth.

*I live in a farm, I built the house, I have a garden, I plant.... Gardening relaxes me because I love my house and the ground I prepared.*

(ES-II-06)

Trying new things and being challenged made PwD proud of their accomplishments in their present lives.

*I saw a new technique, airbrush and thought ‘wow, that is something I would like to try’ and I asked myself what it would be like to produce cards for Christmas and New Year for example and send them to loved ones.*

(DE-II-07)

People with dementia experienced a feeling of empowerment when they felt in control.

*I prepare everything before starting so that I do not get confused. I stopped doing that and got confused. Now I feel much better, when everything is well prepared.*

(DE-II-15)

*I am ready to do everything to delay the progress of the problems I currently experience and as long as I am aware of that and I keep on looking for solutions, I will be able to go on with my life.*

(ES-II-01)

PwD felt what they called ‘normal again’ when meeting with other PwD and therefore valued attending support groups. They described how they enjoyed each other’s trust, how it gave them a feeling of self-worth and that using their resourcefulness made them capable of supporting another person.

*You let the other feel your respect when the other person copes with something. For example, when he stands up and performs in front of us, a song for example. You also respect him, if he wets his pants. You need to trust each other.*

(DE-II-03)

Comparing themselves to others in the support group also supported the interviewees in reframing their identity and appreciation of their own lives and strengthened their feeling of self-worth.

*I really like to come here because I learn, I remember a lot and I am very satisfied with supporting others and sharing my thought with them.*

(ES-II-04)

## 4. Discussion

One of the strengths of the study presented here is the group of PwD still living at home and coming from two European countries, namely, Germany and Spain. After receiving the diagnosis, they continued their lives in their familiar surroundings. When interviewed in the MinD project, they described their social interactions that were different from the time prior to the diagnosis of dementia. In summary, participants from both countries covered very similar categories, subcategories and subcategory contents. All the PwD closely linked retaining a sense of usefulness and the feeling of empowerment to social interaction, showing a large congruity between the German and Spanish PwD. They reported disempowering situations, and when asked what would have been supportive, they described their need to be heard and for interactions in reciprocity. The driving forces in their lives were coping with changes related to dementia, staying in control, and specifically, their effort to retain a sense of usefulness through social participation in reciprocity despite the loss of familiar roles and social contacts. The participants reported feelings of usefulness and empowerment when using their resources to support others through social participation. Additionally, participants in this study expressed a feeling of empowerment by starting something new, e.g., learning a new craft technique, going to lectures or visiting new places. In both countries, several interviewees had experienced support groups with other PwD. They associated these encounters with a feeling of normality, being valued and respected, and thereby, being a part of something. When comparing themselves to other support group members, they reported feelings of joy, hope and appreciation of their own lives. The empowering strategy of recalling personal achievements was central to a majority of interviewees and reminding themselves of important events preserved their identity, self-cohesion and sense of self-worth.

It is worth noting that the PwD in this study expressed similar views on usefulness and empowerment to those described in other studies by elderly people and centenarians without dementia [3,22,23], clearly emphasizing the importance of sustaining social contacts and providing the possibility of interacting with the environment for PwD and old people in general. Recently, concepts for supporting PwD have undergone a shift from task-oriented support to a person-centered approach, explicitly focusing on strengthening personal competencies, decision-making and self-worth, reframing the dementia diagnosis from one of ‘disabled’ to ‘enabled’ [24,25,26,27]. The European research group (INTERDEM) explicitly emphasizes that psychosocial interventions should focus on promoting the autonomy, dignity and rights of PwD, and communities should make themselves available to PwD from early diagnosis onwards [28,29,30]. However, the process is very slow, as a recent survey revealed for Europe [26]. The challenge of an increased life expectancy at birth and a demographic shift towards older age also necessitates programmes that promote healthy ageing in the population, as well as programmes that support PwD as one of the major medical concerns in old age. Furthermore, PwD and their caregivers should be considered as dyads with complex and differing needs in many domains that necessitate specific support throughout the disease. The progressive nature of the disease and the associated decline inevitably have negative consequences for informal caregivers, such as difficulties in communication and in understanding each other’s perspectives, leading to the experience of ongoing losses [31].

Our results highlight that age-friendly communities should provide services and infrastructure to promote health in older people [32] and specifically address issues that are important for PwD living in the community and their carers. In practice, this implies that beyond specific programmes for people directly affected by dementia, promoting public awareness to reduce stigma and stress the importance of social participation and inclusion of PwD and their families is essential and should form part of a community action plan for a more collaborative community [33,34,35,36].

### Study Limitations

The participants of the study presented here had received an ICD-10 diagnosis of dementia. However, the recruitment of participants in both countries was neither randomized nor matched for specific parameters, such as age, gender and education. Participants responded to our invitation after hearing about the study. The German and Spanish groups had some differences, e.g., the age of participants and education, but our analyses revealed no differences in the categories or subcategory contents across the two countries. A further limitation was that we had no information on the support the people with dementia had actually received since their diagnosis, and thus, we could not assess how their views compared with their actual experiences of care and support. The generalizability of our results might be limited to those PwD experiencing mild-to-moderate stages of the disease, still living at home and receiving support from family caregivers on a regular basis. Differences may occur with the progression of dementia, though, of course, it would become more difficult to undertake this kind of study in people with more severe impairment.

## 5. Conclusions

In this study, we found that people living with dementia had strong feelings of usefulness and empowerment if they were reinforced by engaging in meaningful activities, interactions in reciprocity through social participation and staying in control as driving forces in their lives. This is consistent with other literature on elderly people without dementia and centenarians demonstrating how important support of social participation is for well-being with increasing age [3,23]. The direct involvement of PwD as spokespeople and educators is necessary and effective, and further work is necessary to investigate ways of offering meaningful activities that avoid disempowering PwD but instead create supportive environments with adequate access and follow the wishes and needs of PwD [36].

## Figures and Tables

**Table 1:** Demographic characteristics of interviewees.

	Germany	Spain
Method	Semi-structured interviews	Semi-structured interviews
Interviewees (n)	6	6
Gender (female/male)	4/2	4/2
Mean age (years—SD)	81 (5.17)	73.2 (6.64)
Living situation		
- Living alone (n)	3	3
- Living with partner, family (n)	3	3
Education:		
- Low	1	3
- Middle	2	0
- High	3	3

**Table 2:** Coding tree.

Category	Subcategories	Themes
1. Experiencing changes in personal life and coping with changes in life	Changes in cognitive abilities	Performing less well in familiar activities Memory problems Orientation problems Difficulties in following and participating in a conversation
	Changes in emotions	Feeling of sadness Feeling insecure Feeling anxious Feeling of not being understood by others Feeling of loneliness
	Loss of familiar roles	Changes in the parenting role Changes in marital relationship
	Loss of autonomy and self-determination	Not being left alone Be told what to do Not being involved in decision making Being a burden to others Being dependant on others Immobility due to the loss of driver’s licence
	Loss of social contacts	Not being invited to events any more by friends Seeing less of the family Feeling of exclusion
	Adaptive coping strategies	Understanding the caregiver’s reaction and decision-making Asking others for support
	Non-adaptive coping strategies	Avoidance of familiar situations Withdrawal from familiar situations Denial of symptoms and changes due to disease
2. Retaining a sense of usefulness	Experiencing social participation	Importance of making oneself useful again Being part of something
	Experiencing a sense of self-worth	Feeling of being needed Feeling of closeness
3. Feeling empowered	Proudly recalling lifetime achievements	Bringing up children Performance in their job Performance in a hobby
	Proud of accomplishments in the present life	Trying new things Helping the family Being supportive of other people with dementia
	Being in control	Actively seeking and accepting help Preparing oneself for specific situations
	Experiencing a sense of self-worth	Being needed Being valued Feeling one’s identity and self-cohesion Feeling of normality and respect when meeting other people with dementia

## Data Availability

The data presented in this study are available on request from the corresponding author.
